# Computational and Chemical Analysis of the Oxygen Atom Transfer Process of a Dioxo–Molybdenum Complex Incorporated into a Modified UiO-67 (Zr/Ti)

**DOI:** 10.1021/acsorginorgau.5c00097

**Published:** 2026-03-24

**Authors:** Laura Milena Valdivieso Zárate, Ernesto David González Cruz, Kerry Jeffry Wrighton-Araneda, Katherine Paredes-Gil, David Contreras, Fernando Martínez Ortega

**Affiliations:** † Centro de Investigación en Catálisis (CICAT), 28014 Universidad Industrial de Santander, Bucaramanga 680002, Colombia; ‡ Escuela de Ingeniería de Materiales (EIMAT), 28006 Universidad del Valle, Cali, Valle del Cauca 760001, Colombia; § Centro Integrativo de Biología y Química Aplicada (CIBQA), 28042 Universidad Bernardo O’Higgins, Santiago 8370853, Chile; ∥ Departamento de Química, 28092 Universidad Tecnológica Metropolitana, Santiago 8330378, Chile; ⊥ Instituto Universitario de Investigación y Desarrollo Tecnológico (IDT), Universidad Tecnológica Metropolitana, Santiago 8940573, Chile; # Departamento de Química Analítica e Inorgánica, 28056 Universidad de Concepción, Concepción 4030000, Chile

**Keywords:** MOFs, UiO-67, dioxomolybdenum complex, catalysis, ab initio, XPS, EPR

## Abstract

In the present work, a combined theoretical–experimental approach was employed to elucidate the structure of a modified UiO-67 (Zr/Ti) MOF functionalized with MoO_2_Cl_2_ through a three-step process and to propose a detailed mechanism for the oxygen atom transfer reaction. Experimental characterization techniques (X-ray diffraction, X-ray photoelectron spectroscopy, and electron paramagnetic resonance (EPR)) were integrated with periodic and cluster-based density functional theory calculations to validate the synthesis and to model the catalytic behavior. EPR data confirmed the formation of Mo^(V)^ species during the catalytic cycle, supporting the intermediates proposed by theoretical studies. This synergistic strategy enabled a deep understanding of both the synthesis process and the catalyst’s function, demonstrating how theory-guided design and experimental validation can drive the development of next-generation MOFs with tunable reactivity and scalable application potential.

## Graphical abstract

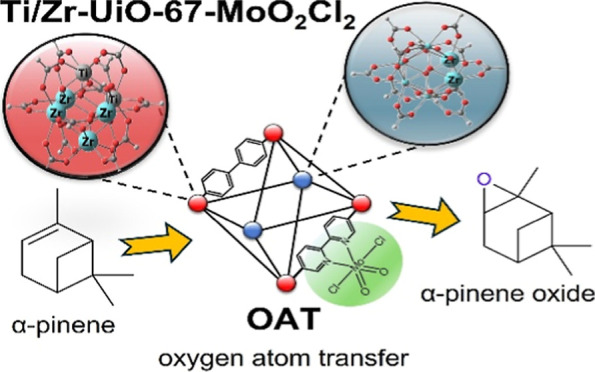

## Introduction

Metal–organic frameworks (MOFs) are nanostructured crystalline compounds formed by metallic units, either isolated atoms or clusters, linked by organic ligands to create one-, two-, or three-dimensional hybrid structures. MOFs stand out for their high porosity and extensive surface area. Their surface area is greater than 1000 m^2^/g.^1^ This positions MOFs as a class of microporous materials with tunable internal properties that are superior to those of mesoporous oxides, zeolites, and carbonates. The diversity of metals and organic molecules that can be used to synthesize MOFs gives them remarkable structural versatility. This versatility enables the design of MOFs with specific physicochemical properties for applications such as gas storage and separation, drug delivery, catalysis, luminescence, water treatment, and CO_2_ capture.^2,3^ The choice of metal and ligand is crucial in determining the structure, stability, and functionality of the material. The metal’s coordination preference influences the number and arrangement of ligands, shaping the pore size and shape. These aspects are crucial for the adsorption capacity and strength of the metal–ligand bond.^4^

UiO-67-type MOFs have become a popular platform for catalytic and adsorption applications due to their high structural stability, precise pore configurations, and ability to be functionalized. These MOFs are composed of Zr_6_O_4_(OH)_4_ clusters linked to ligands such as biphenyl-4,4′-dicarboxylic acid (**bpdc**) and 2,2′-bipyridine-5,5′-dicarboxylic acid (**bpydc**). These clusters generate tetrahedral and octahedral spaces (approximately 7 and 13 Å, respectively) in a 2:1 ratio, favoring the adsorption of gases such as H_2_, CH_4_, and CO_2_.^5–7^

In order to enhance the photocatalytic performance, titanium (Ti) was introduced into the metallic nodes, partially replacing zirconium (Zr) without compromising the network architecture. This modification combines the high thermal stability of Zr with the photoactivity of Ti, optimizing electron transfer under visible light and reducing the HOMO–LUMO gap.^2,8^ The catalytic complex *cis*-[Mo^(VI)^O_2_]^2+^ can be anchored to this structure, mimicking the active sites of molybdenum enzymes, such as sulfite oxidase. This enables oxygen-atom-transfer (oxygen atom transfer (OAT)) reactions—including epoxidation, olefin hydroxylation, and alcohol oxidation—which in turn broadens the catalytic capabilities of the hybrid system.^9–11^

OAT in molybdenum complexes enables the selective oxidation of organic compounds. The metal-oxo bonds of the complex, designated as M=O, can change the configuration depending on the presence and nature of the ligands and the coordination stereochemistry. However, M=O groups are stabilized with metal centers in a highly oxidized state. The formal charge on the metal reduces the basicity of the oxo ligand, so the groups resist nucleophilic attack under normally accessible experimental conditions.^9^

Previously published work experimentally synthesized a series of UiO-67­(Zr) structures by modulating the bpydc/bpdc molar ratio (33:67, 50:50, 60:40, and 0:100). This work confirmed their cubic (Oh, *Pn*3̅*m*) crystal system and spherical morphology by using X-ray diffraction (XRD). The effect of formic acid as a modulator was attributed to this outcome. The BET surface area increased as the bpydc ratio increased (1371–1932 m^2^/g). Also, the incorporation of Ti (66 mol % with respect to Zr) reduced the surface area by ∼200 m^2^/g due to differences in coordination between O–Zr and O–Ti. Subsequently, a dioxo-Mo^(VI)^ complex was anchored on the bpydc ligands, with contents ranging from 2.63% to 4.53% Mo. The complex exhibited excellent catalytic performance in the selective oxidation of α-pinene via UV–vis-activated OAT. The system achieved 95% conversion to epoxide in 10 h, decreasing slightly to 85% after three cycles, with an average molar efficiency of 3:1 (product/Mo).^7^

However, understanding the structure–function relationship of such hybrid systems remains a challenge, particularly in terms of identifying the nature of the active species, their stability under catalytic conditions, and the mechanistic pathways involved in the OAT cycle. To address these gaps, theoretical methods such as density functional theory (DFT), in combination with advanced spectroscopic techniques (X-ray photoelectron spectroscopy (XPS), XRD, and electron paramagnetic resonance (EPR)), provide powerful tools to elucidate reaction mechanisms and validate the formation of intermediates.

In this context, the present work aims to elucidate the structure and the mechanism of the modified UiO-67 (Zr/Ti) MOF functionalized with MoO_2_Cl_2_ evaluated in the OAT process of the epoxidation of α-pinene, using XPS and DFT calculations. Based on the crystallographic structure proposed, we build a cluster model of [Zr_4_Ti_2_O_8_(OOCH)_12_] to understand the atomic rearrangement when Ti is substituted by Zr in the UiO-67 (Zr/Ti) unit. Starting of this structure, three different conformations for the (Ti/Zr)-UiO-67-(bpdc,bpydc)-MoO_2_Cl_2_ model were analyzed in searching for a catalytic species that allow to study the mechanism. Finally, to corroborate the catalytic behavior, the intermediate Mo^(V)^ species was identified using EPR spectroscopy. This study provides a comprehensive understanding of the catalytic performance, advancing the rational design of functionalized metal–organic frameworks (MOFs) with potential industrial applications in green synthesis.

## Experimental Section

### Obtaining the MOF (Ti/Zr)-UiO-67-(bpdc,bpydc)-MoO_2_Cl_2_ in Three steps

The preparation of the UiO-67 (Zr/Ti) MOF network with dioxo-molybdenum complexes involves three steps: first, the UiO-67 (Zr)-type MOFs were synthesized (bpdc/bpydc). Second, a postsynthetic exchange of Ti for Zr was performed in the UiO-67 (Zr)-type MOFs (bpdc:bpydc). Finally, the dioxo–Mo complex was anchored to the bpydc ligands of the UiO-67 (Zr/Ti)-type MOFs (bpdc:bpydc).

Step I was performed according to the protocol reported by the University of Oslo,^12^ using biphenyl-4,4-dicarboxylic acid (bpdc) and 2,2′-bipyridine-5,5′-dicarboxylic acid (bpydc) ligands. First, 1.08 g of zirconium^(IV)^ chloride (ZrCl_4_) was added to a volumetric flask. Then, 0.109 mL of water and 180 mL of *N*,*N*′-dimethylformamide (DMF) were added, and the mixture was heated at 100 °C in a sand bath with magnetic stirring until the mixture became turbid. Then, with constant stirring, formic acid and 1 g the corresponding ligands (bpdc-bpydc) in 50:50 molar ratio were added, resulting in a structure composed of six inorganic clusters Zr_6_O_4_(OH)_4_ with square and antiprismatic coordination of each Zr atom with eight oxygen atoms (Figure 1).^7^ Each inorganic cluster is connected to the others by organic ligands, which can be bpdc and bpydc (Figure 1).

Step II consists of the postsynthetic exchange of Zr for Ti in the UiO-67 clusters (see Figure 1). This process was carried out according to Navarro’s protocol.^1^ The process begins by adding of titanium­(IV) chloride tetrahydrofuran complex (TiCl_4_·(THF)_2_) and DMF to an autoclavable graduated Schott flask. Then, the flask was transferred to a steel autoclave lined with a Teflon jacket and 0.050 g of the (Zr)-UiO-67-(bpdc,bpydc) was added. The solution was ultrasonicated for 30 min at room temperature to achieve complete dispersion. The autoclave was placed in an air oven at 120 °C for 6 days, with two manual stirrings per day to maintain the homogeneity of the solution. The resulting solid was recovered by centrifugation and washed 3 times with DMF, 3 times with ethanol, and 3 times with diethyl ether (10 mL each time). It was then dried in an oven at 40 °C for 12 h.

For step III, a MoO_2_Cl_2_(THF)_2_ complex was prepared according to the procedure reported by Leus et al.^13^ with some modifications. A Schlenk line was used to avoid the presence of oxygen and water. Then, 0.150 g of the (Ti/Zr)-UiO-67-(bpdc,bpydc)-MoO_2_Cl_2_ was added and the solution was stirred for 6 h at room temperature. The solid obtained was recovered by centrifugation and washed with acetone to remove the Mo complex. Finally, it was dried under vacuum for 12 h, resulting in the structure as shown in Figure 1.

**Figure 1:**
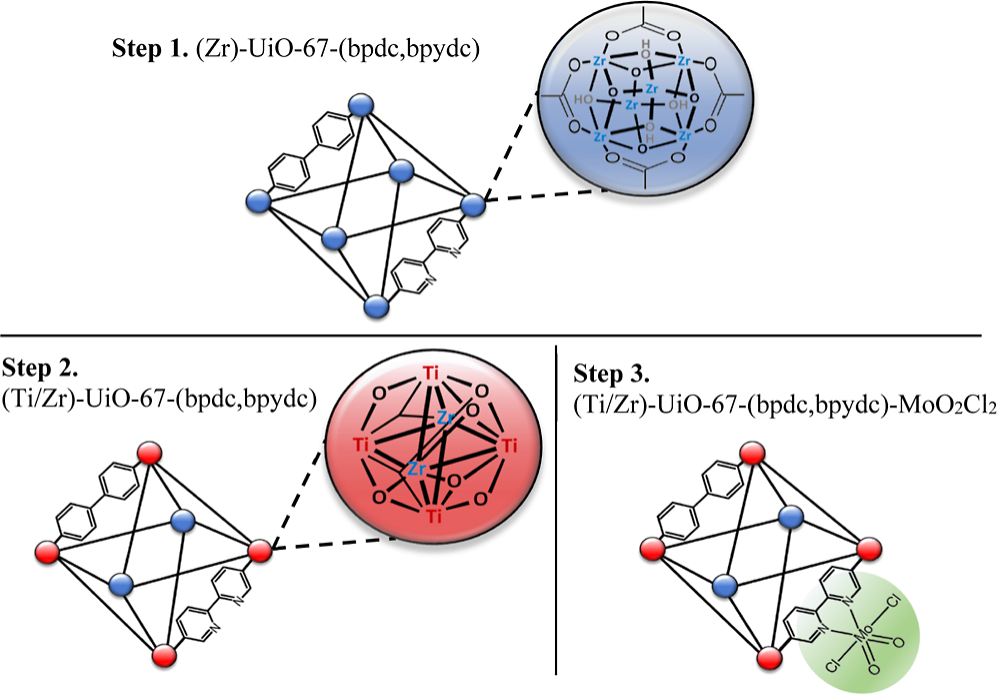
Steps to obtain the MOF-type (Ti/Zr)-UiO-67-(bpdc,bpydc) in the presence of the MoO_2_Cl_2_ complex.

### Experimental Conditions for the Catalytic Reaction

The selective oxidation reaction of α-pinene was carried out as reported by Valdivieso et al.^7^ with some modifications, inside the EPR cell to allow in situ monitoring. 0.015 g of the catalyst (Ti/Zr)-UiO-67-(bpdc,bpydc)-MoO_2_Cl_2_ (3.64% Mo), 0.00271 mL of monoterpene, and 0.030 mL of acetonitrile (solvent) were used with a molar ratio of monoterpene:Mo complex of 3:1. The reaction was performed in steps, each 60 min, with monitoring every 15 min: step 1: UV radiation (350 nm) and an Ar atmosphere; step 2: dark and O_2_ atmosphere; and step 3: UV irradiation (350 nm) and an Ar atmosphere.

### Characterization Techniques

The surface analysis of the MOFs (Ti/Zr)-UiO-67-(bpdc,bpydc)-MoO_2_Cl_2_ during the different synthesis steps was performed by XPS, using an XPS/ISS/UPS surface characterization platform built by SPECS (Germany), equipped with a PHOIBOS 150 2D-DLD energy analyzer belonging to the Surface Science Laboratory (SurfLab) of the Universidad Industrial de Santander (UIS). During the analysis, the pressure in the chamber was approximately 1 × 10^–9^ Pa. A monochromatic Al Kα X-ray source (FOCUS 500) operating at 100 W was used for the measurements. The pass energy of the hemispherical analyzer was set to 100 eV for general spectra and 15 eV for high-resolution spectra. Surface charge compensation was controlled by a flood gun (FG 15/40 PS FG 500) which was operated at 50 μA—1.8 eV. The reference scale was calibrated by setting the adventitious carbon C–H to 284.8 eV. The XPS data were analyzed using CasaXPS software.

Electronic paramagnetic resonance (EPR) was analyzed with a spectrometer belonging to the Research Group of Fundamentals and Applications of Advanced Processes of the Universidad de Concepción (UdeC) that consisted of a cell fixed inside the Bruker ER 4119HS cavity of the Bruker EMX microspectrometer operating in the X-band. The microwave power of the EPR was set to 2000 mW, and the modulation frequency was 100 kHz. The receiver gain was set to 10 dB. The quantification of the amount of spin was performed using the quantification algorithm integrated in Xenon software according to the manufacturer’s instructions and considering the technical parameters of the cell used.

XRD analysis was performed on a Bruker diffractometer model D8 Advance, with DaVinci geometry, Cu Kα radiation (λ = 0.15406 nm), a linear detector, and 0.6 mm divergence slit located at the X-ray Laboratory of the Universidad Industrial de Santander (UIS).

### Computational Methodology

Quantum chemical modeling through DFT framework was carried out to study the influence of the electronic structure on the structural, electronic, stability, and catalytic properties of the modified UiO-67 (Zr/Ti) MOF reported by Valdivieso-Zárate and co-workers.^7^

For the construction of periodic structural models based on the modified UiO-67 (Zr/Ti) MOF structure, three databases were revised: Material Project, Crystallography Open Database, and Cambridge Structure Database.^14–16^ Thus, three crystal structures were used to construct periodic models based on (Ti/Zr)-UiO-67-(bpdc,bpydc)-MoO_2_Cl_2_ (WIZMAV01 [CSD: 1018032; CODID: 7112002]; XIVTED [CSD: 968930; CODID: 7112002]; BIMDOQ02 [CSD: 1997752]). Crystal structure modifications based on UiO-67 were performed using CrystalMaker 10.8, VESTA3.5.8, and Chemcraft visualizers.^17,18^ The models are described in Figure 2.

**Figure 2:**
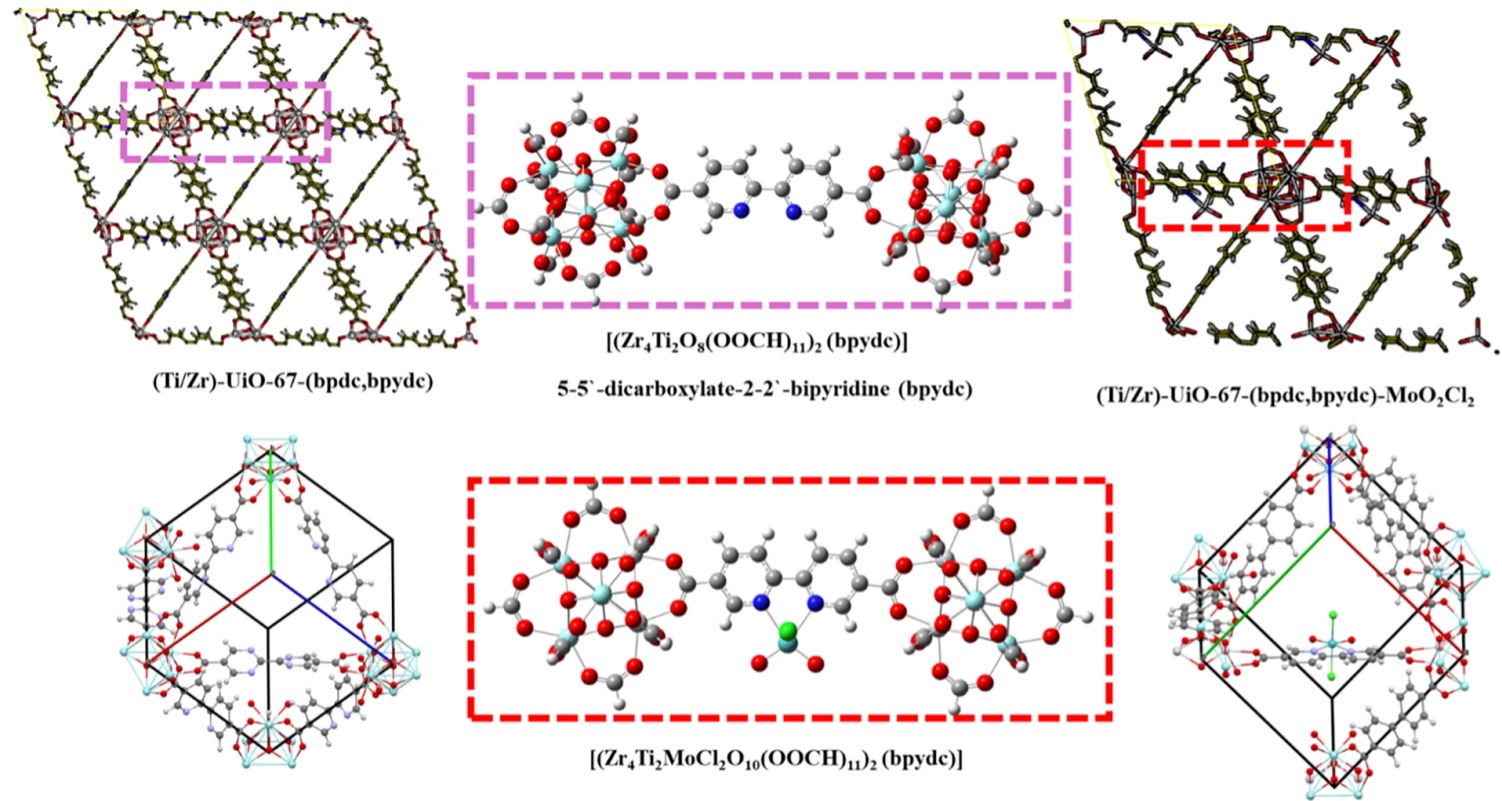
Design based on crystal structures for Ti-doped structures, and MoO_2_Cl_2_ for the construction of computational models based on UiO-67 structures.

Periodic state models were developed using CP2K^19^ to evaluate solid-state properties using a PBE functional while treating all atoms with Gaussian plane waves for the basis set and norm-conserving Goedecker-Tetter Hutter for pseudopotentials, DZVP-MOLOPT-PBE-GTH and GTH,^20,21^ respectively. Electronic energies were calculated using the QuickStep method at a medium threshold level for SCF evaluations including dispersion correction by the D3BJ method.^22–24^ Cell-fixed relaxations of the molecular structure were carried out for all periodic systems because powder XRD showed no significant changes by the synthetic procedure. The relaxed crystal structures were then compared to the experimental diffractogram to validate the structural models.

Furthermore, to build a cluster model of (Ti/Zr)-UiO-67-(bpdc,bpydc)-MoO_2_Cl_2_, we start from the crystallographic structure. First, an octahedral cluster [Zr_6_O_8_(OOCH)_12_] with Zr and Ti atoms at the vertices linked by poly­(carboxylates) was truncated with the aim of finding the atomic positions of the Ti atom when it is exchanged by Zr in the cluster. Next, a model with two cluster units [(Zr_4_Ti_2_O_8_(OOCH)_12_)_2_(bpydc)] is built and the coordination of the bipyridine (bpydc) to the cluster of the Zr/Zr, Zr/Ti, and Ti/Ti bonds is studied. Finally, the dioxo–Mo complex [Mo^(VI)^O_2_Cl_2_] was added to the most stable structure with the aim of obtaining a cluster model of (Ti/Zr)-UiO-67-(bpdc,bpydc)-MoO_2_Cl_2._ This is done by studying the OAT mechanism of the epoxidation of α-pinene.

All the calculations related to the [(Zr_4_Ti_2_ MoCl_2_O_10_(OOCH)_11_)_2_(bpydc)] cluster model have been performed within the framework of DFT using the program GAUSSIAN 16. Optimization geometries in the gas phase of the structures were performed using the exchange–correlation GGA functional, B3LYP, incorporating the D3-Grimme’s dispersion correction. The core electrons of zirconium, titanium, and molybdenum atoms were described by a quasi-relativistic effective core pseudopotential (relativistic effective core pseudopotential) called LANL2DZ, while the rest of the electrons are described by the associated (8s7p6d)/[6s5p3d] basis set. The 6-31G basis set was used to describe carbon, hydrogen, nitrogen, oxygen, and chlorine atoms. The nature of each structure as an intermediate or transition state on the potential energy surfaces, was analyzed by calculating the Hessian matrix in internal coordinates (second derivative of the energy with respect to position). These frequencies were used to determine the thermal contributions to enthalpy (H) and Gibbs free energy (G) by utilizing the harmonic oscillator and rigid rotor model implemented in Gaussian-16 and assuming a temperature of 298 K at standard pressure (1 atm).

## Results and Discussion

### Structural Elucidation of Modified UiO-67 (Zr/Ti)

After synthesis, XPS was used to observe the stepwise formation of the MOF. The focus was on the elemental composition and the local coordination environments associated with the construction of the framework, substitution of the metals, and anchoring of the Mo dioxo complex. Figure 3 shows the high-resolution spectra obtained by XPS for the (Zr)-UiO-67-(bpdc,bpydc) framework obtained in step I, where the presence of the elements that make up the MOFs structure is observed. The C 1s spectrum (Figure 3a) shows four different chemical species. At 284.8 eV, the C=C double bond is present in the molecule related to the bpdc-bpydc aromatic compounds. The C–N single bond at 286.1 eV corresponds to the bpdc or bpydc bond structure.^25^ At 288.1 and 289.4 eV, the O–C=O group and the carbon–carbon double bond associated with the aromatic ligands (π–π component) are present.^25^ The O 1s spectrum (Figure 3b) displays two distinct signals. The peak at 530.2 eV corresponds to the Zr–O single bond present in the clusters, while the peak at 531.8 eV is associated with the O–C bond located at the ligand’s anchoring sites on the cluster.^25,26^ For Zr 3d (Figure 3c), the centered spin–orbit splitting shows two species located at 182.3–184.6 and 183.4–185.7 eV, respectively. These energies correspond to the Zr–O and Zr–OH bonds located in the MOF clusters.^25–27^ Finally, the N 1s spectrum (Figure 3d) shows the presence of N–C and N=C bonds located at 399 and 400.9 eV.^25,26^

**Figure 3:**
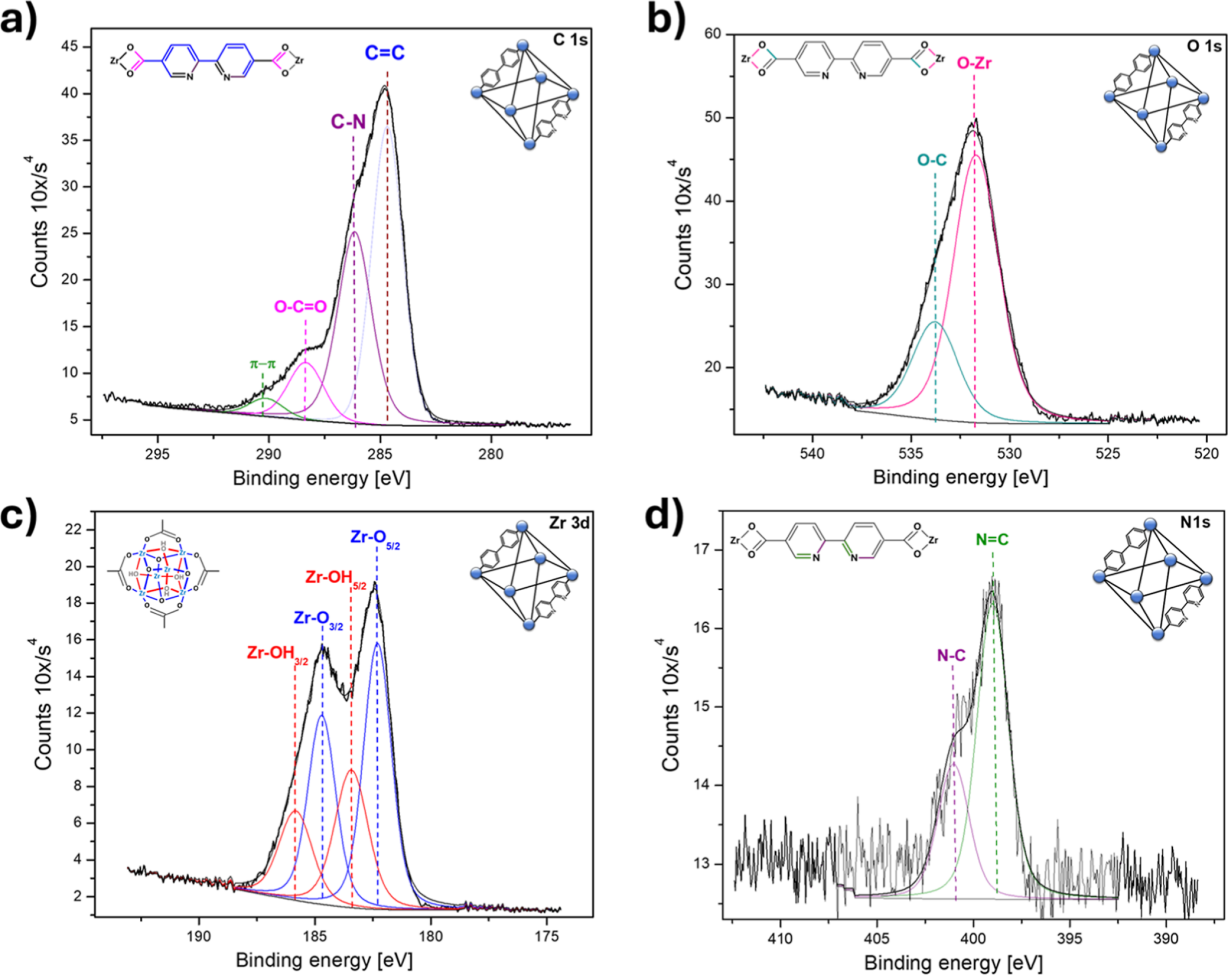
High-resolution XPS spectra of (Zr)-UiO-67-(bpdc,bpydc) MOF after the first synthesis step: (a) C 1s, (b) O 1s, (c) Zr 3d, and (d) N 1s. The characteristic peaks for C–C/C=C, C–N, O–C=O, π–π*, Zr–O, Zr–OH, O–C, and N–C/N=C bonds are observed, confirming the expected chemical composition and coordination environment.

Figure 4 presents the high-resolution XPS spectra of the UiO-67 MOF after (Ti/Zr)-UiO-67-(bpdc,bpydc) exchange (step II), where variations in peak intensities and certain chemical states indicate modifications in lattice coordination resulting from the exchange reaction. The C 1s spectrum (Figure 4a) shows four states at 284.8, 285.1, 286.2, and 288.4 eV, corresponding to the C=C, C=N, C=O, and O=C–O bonds, respectively. These peaks exhibit slight shifts compared with the values from step I, influenced by the substitution of Zr with Ti.^28^ For the O 1s (Figure 4b), we have the presence of the O–Ti/Zr which is due to the bonding between the metal present in the cluster with the respective ligand at 530.2 eV, the O–C at 531.8 eV, and the O–H at 533.4 eV.^29^ Regarding Ti 2p (Figure 4c) in this one Ti–O appears at 458.5 eV, confirming that the exchange reaction in the cluster was successful.^28^ The Zr 3d spectrum (Figure 4d) shows two species at 181.7 and 182.4 eV, corresponding to Zr–O and Zr/Ti–O, respectively, within the MOF clusters. These peaks shift relative to those observed in step I, reflecting changes in ligand coordination caused by the transition metal present in the clusters. Several authors have shown that when different metals interact with the bpdc and bpydc ligands, they alter the coordination environment of the MOF, leading to observable chemical shifts in the XPS spectra.^30^ Finally, the N 1s (Figure 4e) maintains the same behavior as in the sample before the exchange with the presence of two species N=C and N–C located at 399 and 401.7 eV.^31^

**Figure 4:**
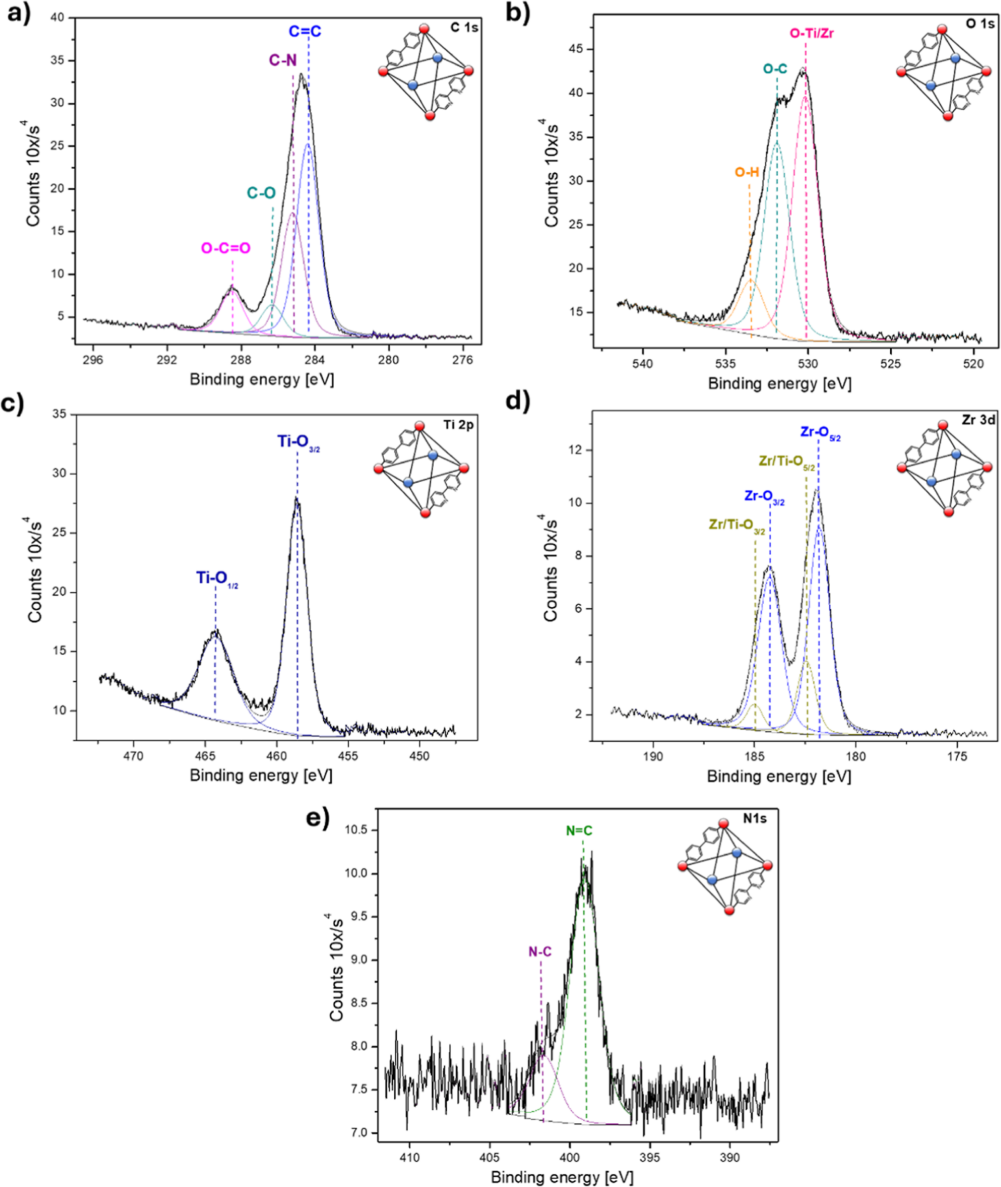
High-resolution XPS spectra of (Ti/Zr)-UiO-67-(bpdc,bpydc) MOF after partial substitution of Zr by Ti: (a) C 1s, (b) O 1s, (c) Ti 2p, (d) Zr 3d, and (e) N 1s. The appearance of Ti–O and mixed Zr/Ti–O signals indicate the successful incorporation of titanium into the metal–organic framework.

The XPS results of the MOF after step III, (Ti/Zr)-UiO-67-(bpdc,bpydc)-MoO_2_Cl_2_, are shown in Figure 5. The C 1s spectrum (Figure 5a) shows changes with respect to step II with the appearance of the C–C bond at 283.2 eV. The remaining signals correspond to the C=C, C=N, and C=O bonds, with binding energies similar to those observed in previous steps (284.8, 286.3, and 288.6 eV). In the O 1s spectrum (Figure 5b), the two species identified in step II remain unchanged: Zr/Ti–O at 530.1 eV and O–C at 531.8 eV. Likewise, the Ti 2p spectrum (Figure 5c) shows the Ti–O species at 458.3 Ev. For Mo 3d (Figure 5d), there is a species at 233.2 eV corresponding to Mo–O which confirms the successful anchoring of the Mo complex to the bpydc ligands of the MOF.^32^ Zr 3d (Figure 5e) presents two species located at 181 and 182.3 eV corresponding to Zr–O and Zr/Ti–O. Finally, N 1s (Figure 5f) presents two species N=C and N–C located at 399 and 401.6 eV previously identified in step II and step I.

**Figure 5:**
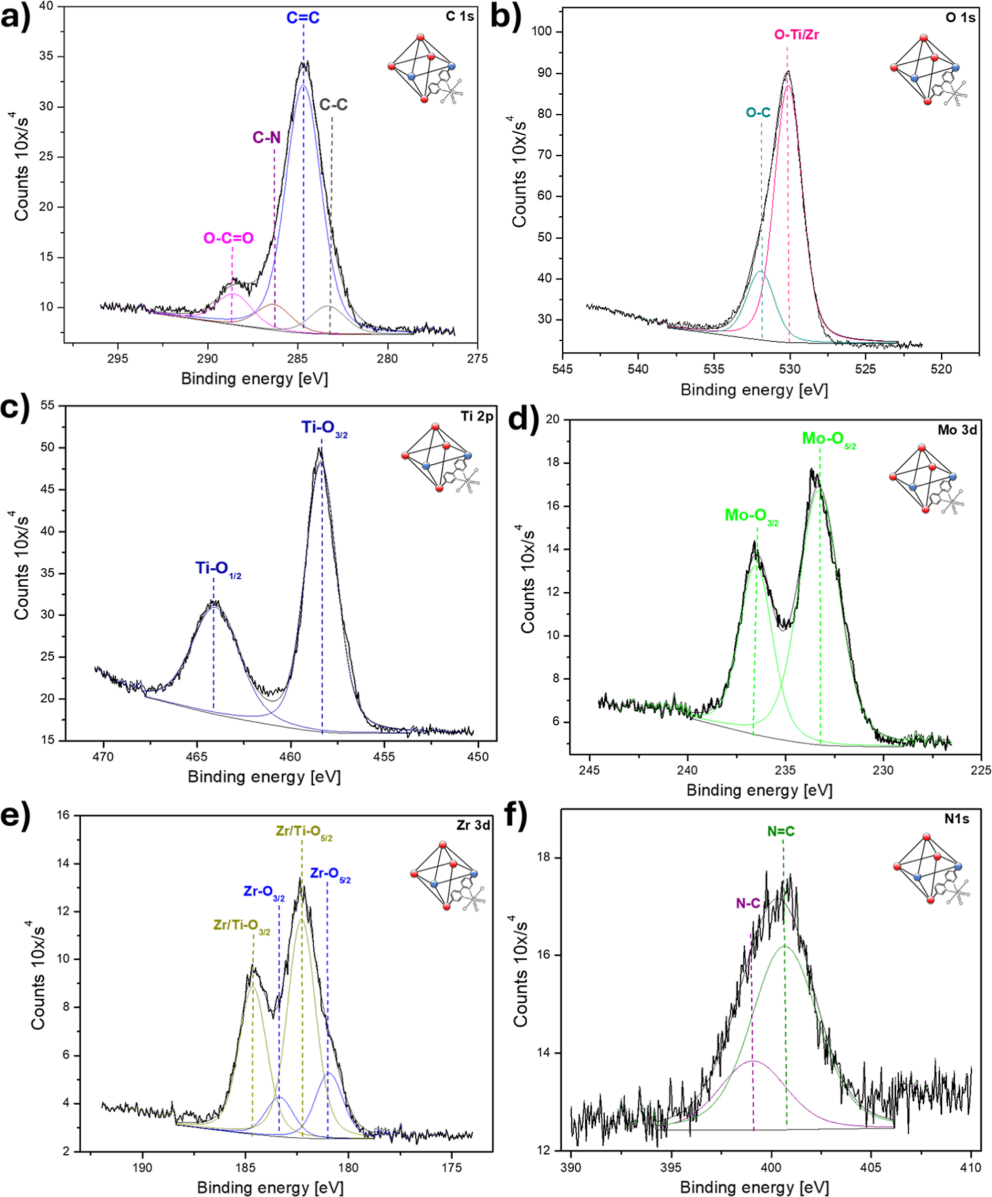
High-resolution XPS spectra of (Ti/Zr)-UiO-67-(bpdc,bpydc)-MoO_2_Cl_2_ MOF after incorporation of the dioxomolybdenum complex: (a) C 1s, (b) O 1s, (c) Ti 2p, (d) Mo 3d, and (e) Zr 3d. The characteristic Mo–O signals demonstrate the successful anchoring of the catalytic complex within the material.

The variations in peak intensities and the appearance or disappearance of species between synthesis steps can be explained by structural changes in the MOF, especially due to the introduction of new elements, such as Ti and Mo. These changes modify the local coordination environment around the Zr centers, causing shifts in binding energies and formation of new XPS signals. Specifically, assigning the Zr 3d spectra as Zr–O/Zr–OH in the initial stage and as Zr–O/(Zr/Ti–O) after Ti incorporation is consistent with the formation of mixed Zr–O–Ti bonds. This behavior is well documented in the literature, where partial substitution of Zr by Ti in MOFs and related oxides generates new coordination states and shifts the Zr 3d binding energies due to changes in the electronic environment and lattice strain.^6,26,28,30,31^ These effects will be further confirmed through DFT calculations, which show good agreement with the experimental observations reported in this work.

Additionally, XPS survey quantification of the final material (step III) yields a surface metal ratio of Zr/Ti/Mo = 1.00/2.09/1.97 (normalized to Zr). This ratio is consistent with the partial substitution of Zr by Ti within the inorganic clusters and the postsynthetic anchoring of the Mo dioxo complex on bpydc sites, as expected for a surface-functionalized UiO-67 framework.

Subsequently, the exploration of crystallographic databases structural information related to the Zr-UiO-67 structure has occurred since there is no crystal structure of partial substitution between bpdc and bpydc. For example, both Zr-UiO-67-bpdc and Zr-UiO-67-bpydc structures (WIZMAV01 and XIVTED, respectively) show face-centered cubic lattice, where the former displays a *Fm*3̅*m* space group while the latter shows a subgroups *Fm*3̅. Thus, both space groups are associated indicating that both structures shared several symmetry operations mirror planes, rotoinversion axes, and inversion, nevertheless, *Fm*3̅*m* is more symmetric than *Fm*3̅ since in crystallographic terms. Therefore, *Fm*3̅ is a subgroup of *Fm*3̅*m* with half as many symmetry operations because *Fm*3̅*m* has an extra set of mirror-related operations. In this specific case, both structures display similar cell parameters, with differences of about 1.2% in the cell edge length and/or ∼3.7% in cell volume. In this sense, we have developed a reduced structural model since the original structures contain ∼632 atoms for each cell, while our model has only 154 atoms, decreasing substantially the computational cost. To validate the correspondence between model and reported crystal structures, we have proved that WIZMAD01 and XIVTED present similar power diffractograms, while our model retains the main structural features allowing to reproduce diffractogram as shown in Figure 6a. A suitable match between experimental and simulated diffractograms was found, then 2θ angles (until 20°) are listed for simulation (experimental): 5.76° (5.72°), 6.66° (6.62°), 9.42° (9.39°), 11.04° (11.02°), 11.54° (11.51°), 13.34° (13.30°), 17.31° (17.34°), 19.75° (19.76°), and 20.06° (20.01°), among others.

**Figure 6:**
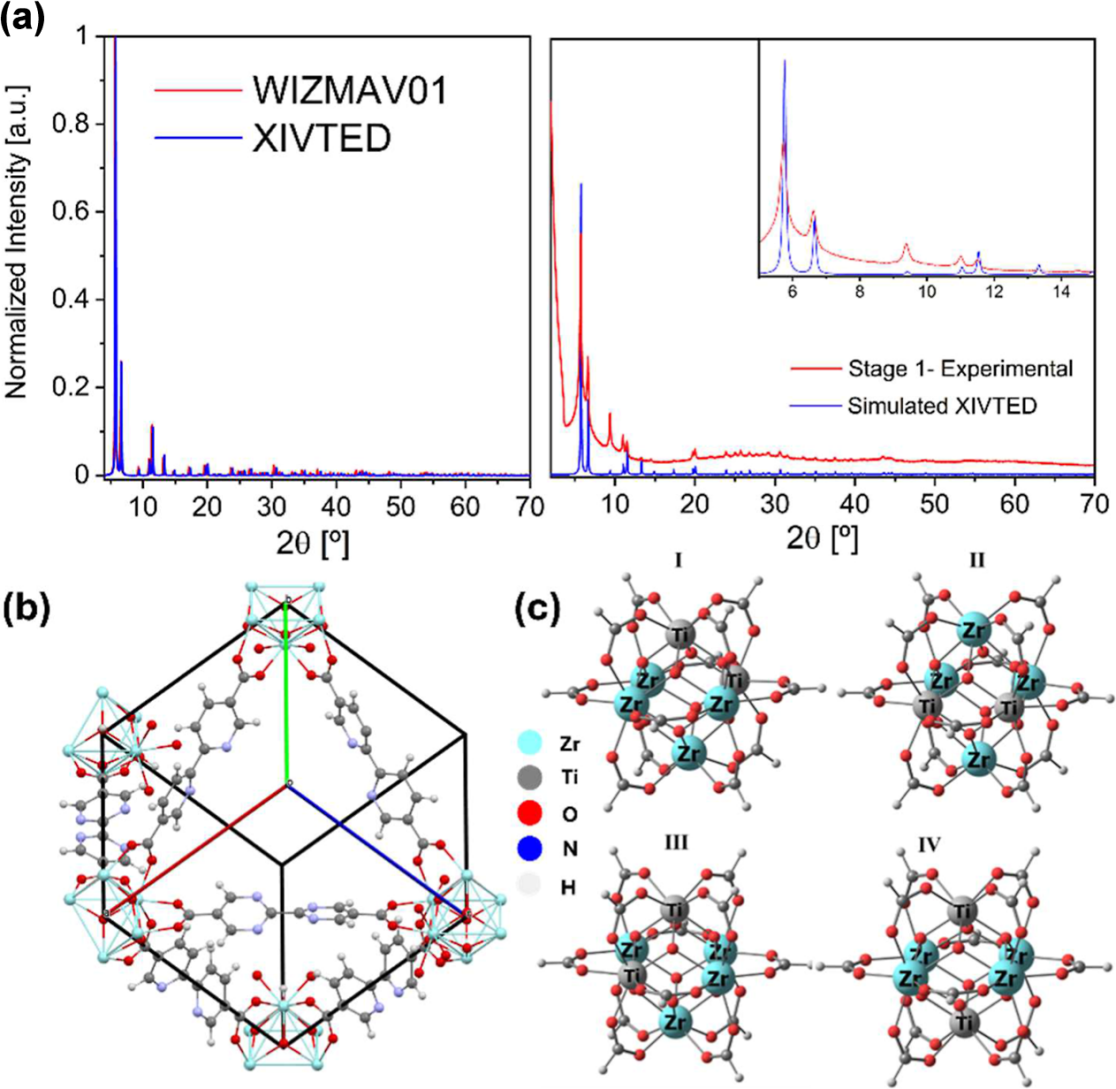
(a) Superposition between experimental and the simulated powder diffractograms of crystal structure XIVTED, WIZMAV obtained from the (Zr)-UiO-67-(bpdc-bpydc). (b) Simulated structural model of (Zr)-UiO-67-(bpdc,bpydc). (c) Four isomers of the truncated cluster model for Ti substitutional models.

The first objective is to obtain the MOF structure in which Ti replaces 33% of the metal centers. To achieve this, we evaluated the most favorable conformation/isomer for Ti-substitution in the truncated cluster model, considering configurations I–V shown in Figure 6b. The stability of each isomer was assessed using the relative energy (Δ*E*_rel_), calculated with respect to the most stable isomer, the I-(Zr/Ti) cluster, as follows

1 $${\Delta E}_{rel}=E_{isomer}-E_{I-\left(Zr/Ti\right)}$$

where *E*_isomer_ and *E*_I_ are the total energy of each isomer and the energy of the I-(Zr/Ti) cluster, respectively. In this sense, ΔE_rel_ describes the energetic destabilization of each isomer in comparison to the most stable isomer and facilitates its determination. Note that I– III by symmetry should be same but present small differences which are key for identifying the Zr/Ti rearrangement into the cluster. In this context, the I-(Zr/Ti) cluster is the most stable where the distance between Ti–Ti displayed the lower length (3.297 Å) demonstrating that side Ti-substitutional doping provide the best conformation since I-, II-, and III-(Zr/Ti) isomers are closer in stability varying up to 3.6 kcal/mol for III-(Zr/Ti) (Table 1). Conversely, the IV-(Zr/Ti) cluster showed a transversal Ti substitutional doping where the Ti–Ti distances reach the larger distance, and the Ti–Zr–Ti angles adopted a right-angle disposition. This evidence is explained in terms of Ti–O bond lengths, which are shorter than Zr–O ones, for example, Ti–O average bond length oscillates between 1.94 and 1.96 Å^33^ while Zr–O 2.09 to 2.37 Å.^34,35^ Thus, lateral Ti-substitution generates a local distortion of the structure sharing similar coordination spheres and bond lengths, indicating the most stable isomer.

**Table 1:** Summary of the Relative Energy (Δ*E*_rel_), Ti–Ti Distances, and Ti–Zr–Ti Angle for the [Zr_4_Ti_2_O_8_(OOCH)_12_] Cluster

isomers cluster model **I**–**IV**	Δ*E*_rel_ (kcal/mol)	distances **Ti**–**Ti** (**Å**)	angles **Ti**–**Zr**–**Ti** (**°**)
**I**-(**Zr**/**Ti**) cluster	0.0	3.297	45.2
**II**-(**Zr**/**Ti**) cluster	1.4	4.448	49.2
**III**-(**Zr**/**Ti**) cluster	3.6	3.384	41.7
**IV**-(**Zr**/**Ti**) cluster	10.3	5.043	90.3

Thus, as the lateral Ti-substitution is energetically favored, we built a structural model of (Ti/Zr)-UiO-67-(bpdc,bpydc)-MoO_2_Cl_2_. In this regard, we proposed a representative model of (Ti/Zr)-UiO-67-(bpdc,bpydc)-MoO_2_Cl_2_ which was built based on the mixture of experimental crystal structure of MoO_2_Cl_2_ (BIMDOQ02 [CSD: 1997752]) and the previous (Ti/Zr)-UiO-67-(bpdc,bpydc) model. The purpose of this pictorial model is simply to capture the main structural features of the material, ensuring consistency with previously studied models. This approach is necessary because no crystal structure has been reported for (Ti/Zr)-UiO-67-(bpdc,bpydc)-MoO_2_Cl_2_ due to its high structural complexity and the limited availability of similar (Ti/Zr)-UiO-67-(bpdc,bpydc) models. Figure 7a shows an acceptable correlation between the powder diffractogram experimental and simulated from the pictorial model built from the cluster model results, as displayed on Figure 7b. Therefore, to understand the M­(Zr or Ti)–O coordination, three possible (Zr/Zr)-, (Ti/Zr)-, and (Ti/Ti)-cluster were built to explain the stability of (Ti/Zr)-UiO-67-(bpdc,bpydc) when is incorporated MoO_2_Cl_2_ catalytic sites (Figure 7c) as was demonstrated in our previous work.^7^

**Figure 7:**
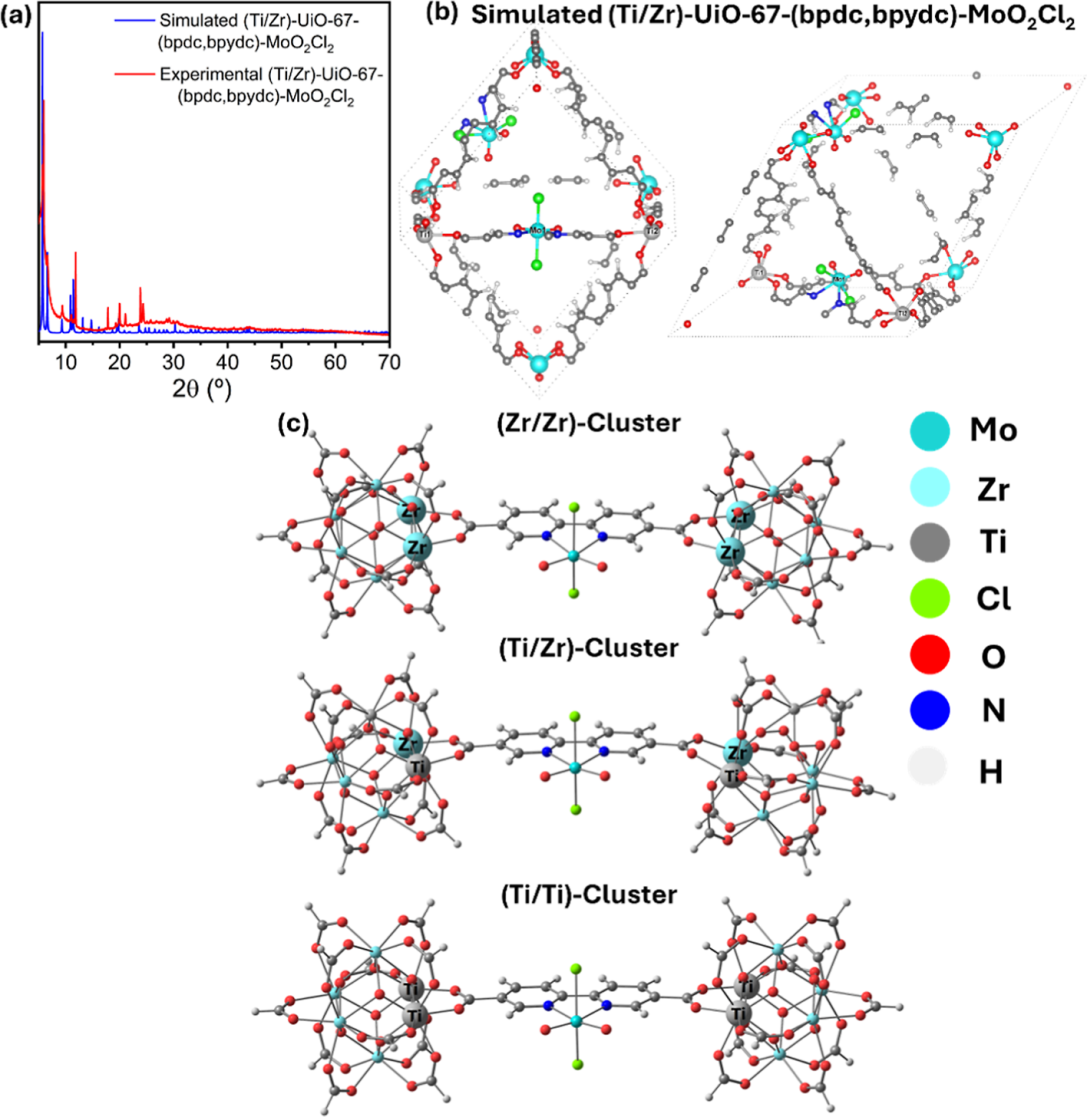
(a) Superposition between experimental and the simulated powder diffractograms of (Ti,Zr)-UiO-67-(bpdc,bpydc)-MoO_2_Cl_2_ structure. (b) Simulated structural model of (Ti,Zr)-UiO-67-(bpdc,bpydc)-MoO_2_Cl_2_. (c) Isomer models of (Zr/Zr) cluster, (Ti/Zr) cluster, and (Ti/Ti) cluster.

Table 2 shows that the (Ti/Ti) cluster is the most stable structure, followed by the (Zr/Ti) and (Zr/Zr) clusters. The Ti–O bond distances in the (Ti/Ti) cluster are shorter than the Zr–O distances in the (Zr/Zr) cluster and the Zr–O/Ti–O distances in the (Zr/Ti) cluster, indicating that the Ti–O interaction is the strongest and most stabilizing, consistent with the energetic results. Moreover, these distances are similar to those reported for other M-O bonds.^36^ On the other hand, the MoO_2_Cl_2_ unit exhibits characteristic structural parameters such as Mo–O, Mo–N, and Mo–Cl bond lengths and O–Mo–O angles, with values of 1.72, 2.32, and 2.39 Å and 106.5°, respectively. Comparison with crystallographic data shows that the MoO_2_Cl_2_ unit is preserved, matching values reported for a similar structure, (H_2_pytz)­[MoO_2_Cl_2_(pytz)] [Hpytz = 5-(2-pyridyl)­tetrazole; GASPUP (CSD: 2118463)][Bibr ref10] where the corresponding bond lengths are 1.69, 2.29, and 2.37 Å, and the angle is 106.1°. Therefore, the mechanistic analysis presented in the next section is carried out using the most stable (Ti, Zr)-UiO-67-(bpdc,bpydc)-MoO_2_Cl_2_ cluster identified in this study.

**Table 2:** Summary of Relative Energy (Δ*E*_rel_) and M-O Distances for (Ti/Zr)-UiO-67-(bpdc,bpydc)-MoO_2_Cl_2_ Cluster

isomers cluster model **I**–**IV**	Δ*E*_rel_ (kcal/mol)	distances **M**-**O** (**Å**)
(Zr/Zr) cluster	8.1	2.208, 2.333, 2.208, 2.233
(Zr/Ti) cluster	7.7	2.185, 2.128(Ti), 2.170, 2.197(Ti)
(Ti/Ti) cluster	0.0	2.035, 2.124, 2.035, 2.124

These results show that the simulated structures of (Zr/Zr)-UiO-67-(bpdc,bpydc) and (Ti/Zr)-UiO-67-(bpdc,bpydc) are stable and can indeed be present in the crystallographic framework, as supported by XPS analyses. This demonstrates a strong correspondence between the theoretical models and experimental spectra. This correspondence reinforces the structural reliability of the proposed models and increases the confidence in the accuracy of the simulation approach.

### Mechanistic Elucidation of the OAT Process of the Epoxidation of α-Pinene

A modified UiO-67 (Zr/Ti) MOF containing a dioxo–Mo complex incorporated into the linker was evaluated as a heterogeneous catalyst for the epoxidation of α-pinene. The experimental results revealed a 1:3 ratio between the MOF and the epoxide formed, which served as the basis for constructing the DFT reaction mechanism.^37^ In our study, we used gas chromatography coupled with flame ionization detection and mass spectrometry to identify and quantify the reactant (α-pinene) and product (α-pinene oxide). Chromatographic analyses revealed a single, well-defined signal corresponding to epoxide, as shown in Figure 8.

**Figure 8:**
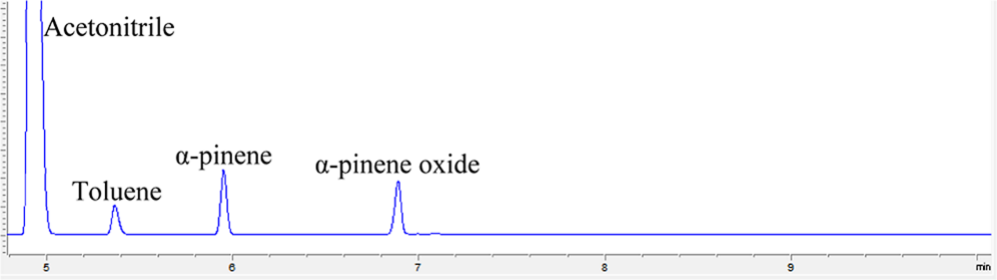
Chromatographic analyses of products during epoxidation of α-pinene by OAT from dioxo–Mo complex incorporated into UiO-67 (Zr/Ti) MOF.

To verify the OAT process from the Mo center to the α pinene molecule, we designed a three-step sequence involving controlled irradiation and atmospheric conditions. We used a solution containing 0.00271 mL (x-mol) of α-pinene, and 0.030 mL (x-mol) of acetonitrile was used in all steps.

In step 1 (UV irradiation at 350 nm under Ar), the Mo=O group of the anchored complex transferred one oxygen atom to α-pinene, yielding a 1:1 ratio of Mo to α-pinene oxide. In step 2 (dark, O_2_ atmosphere), the Mo center was reoxidized, as confirmed by XPS and FT IR, which revealed the reappearance of the Mo–O–O peroxo species and the formation of a Mo=O bond. In step 3 (UV irradiation at 350 nm under Ar), the peroxo ligand transferred its two oxygen atoms to α-pinene, generating two additional epoxide molecules per Mo center; thus, after completing one full OAT cycle (steps 1–3), a total of three molecules of α-pinene oxide were formed per one Mo center present in the MOF, confirming a 1:3 ratio.

For the OAT reaction, a mechanism based on oxido-peroxide molybdenum^(VI)^ complexes has been proposed.^36^ In general, oxygen-atom transfer to α-pinene can occur either stepwise or in a single step (phase 1), followed by reoxidation by O_2_ to form an oxo-peroxo Mo species (phase 2), which then continues the catalytic cycle (phase 3) (see Figure 9).

**Figure 9:**
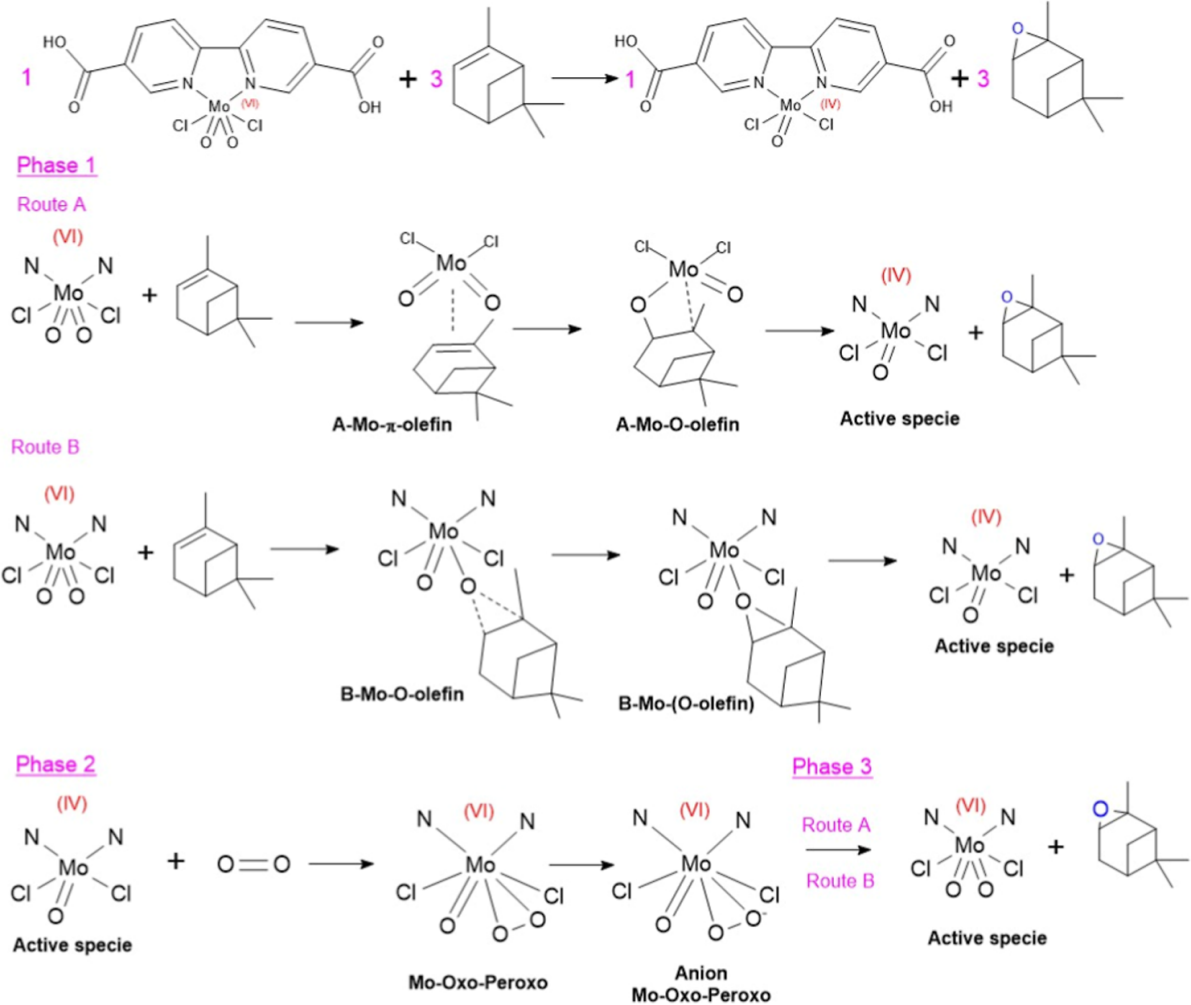
Proposed OAT mechanism using the modified UiO-67 (Zr/Ti) MOF. Phase 1: one oxygen atom is transferred from the dioxo-Mo center to α-pinene. Phase 2: reoxidation of the Mo^(V)^ center by molecular oxygen to the former Mo–O–O peroxo species. Phase 3: OAT from the molybdenum oxo-peroxo center to α-pinene.

In phase 1, the first route (route A) starts with an olefin attack on the metal center, generating a p-coordination intermediate that is transformed into a four-membered alkyl peroxide metal cycle, giving rise to the active species and the epoxide. The second olefin pathway occurs through a direct attack on the double bond of the olefin at one on the oxygens of the MoO_2_Cl_2_ group (route B). Thus, the proposed OAT mechanism (Figure 9) will be experimentally analyzed and validated to clarify the thermodynamics of the process and to identify key intermediates that help explain how the reaction proceeds.

We examined the OAT process for the epoxidation of α-pinene using the cluster model (Ti/Zr)-UiO-67-(bpdc,bpydc)-MoO_2_Cl_2_ shown in Figure 10. In phase 1, we evaluated all intermediates along routes A and B, while in phase 2, we focused on the formation of the Mo oxo-peroxo species to clarify the role of this species—and its anionic form—in driving the reactivity observed in phase 3. In phase 1, the Gibbs energy values show that the reactants are more stable than the product of step 1, which could be complicated for a catalytic process where formation of active species is key. Nevertheless, the high stability of the intermediates leads to the reaction toward the formation of the α-pinene oxide. In route A, the first intermediate is an **A**-**Mo**-**p**-**olefin** characterized by the presence of p-coordination between the Mo atom and the double bond of the olefin to 3.450 Å. This intermediate is 9.1 kcal/mol more stable than the reactant but 14.1 kcal/mol less stable than the most stable intermediate. This species shows an asymmetric coordination of the olefin to the active center MoO_2_Cl_2_ with distances of 2.570 and 2.966 Å between the oxygen of the oxo group and the carbon atoms of the olefin. Thus, this is not exactly an **A**-**Mo**-**p**-**olefin** because a four-membered alkyl peroxide metallacycle metal cycle was not obtained. Next, an **A**-**Mo**-(**O**-**olefin**) intermediate is generated with shorter distances than the above intermediate, these being 1.866 and 1.656 Å between the oxygen of the oxo group and the carbon atoms of the olefin. These three intermediates showed a longer distance than the reactant correspond to 0.005, 0.100, and 0.447 Å for the Mo–O bond of **A**-**Mo**-**π**-**olefin**, **A**–**Mo**–**O**-**olefin**, and **A**-**Mo**-(**O**-**olefin**), respectively, which suggests that this distance will increase until the rupture. On the other hand, it is important to highlight that the Gibbs energy values for all the intermediates in route B are lower than in route A. Therefore, route B is the most thermodynamically favorable pathway in step 1. It begins with the formation of a **B**–**Mo**–**O**-**olefin** intermediate, produced by a direct attack of the olefin’s double bond on the oxo group of the active Mo center. This interaction creates a symmetric coordination, with distances of 2.912 Å between the oxo oxygen and the olefin’s carbon atoms. The subsequent **B**–**Mo**-(**O**-**olefin**) intermediate shows shorter O···C distances—2.013 and 1.899 Å—relative to the previous species, while the Mo–O bond lengthens, indicating the formation of α-pinene oxide. This symmetry rearrangement lowers the Gibbs energy, making route B thermodynamically more stable than route A.

**Figure 10:**
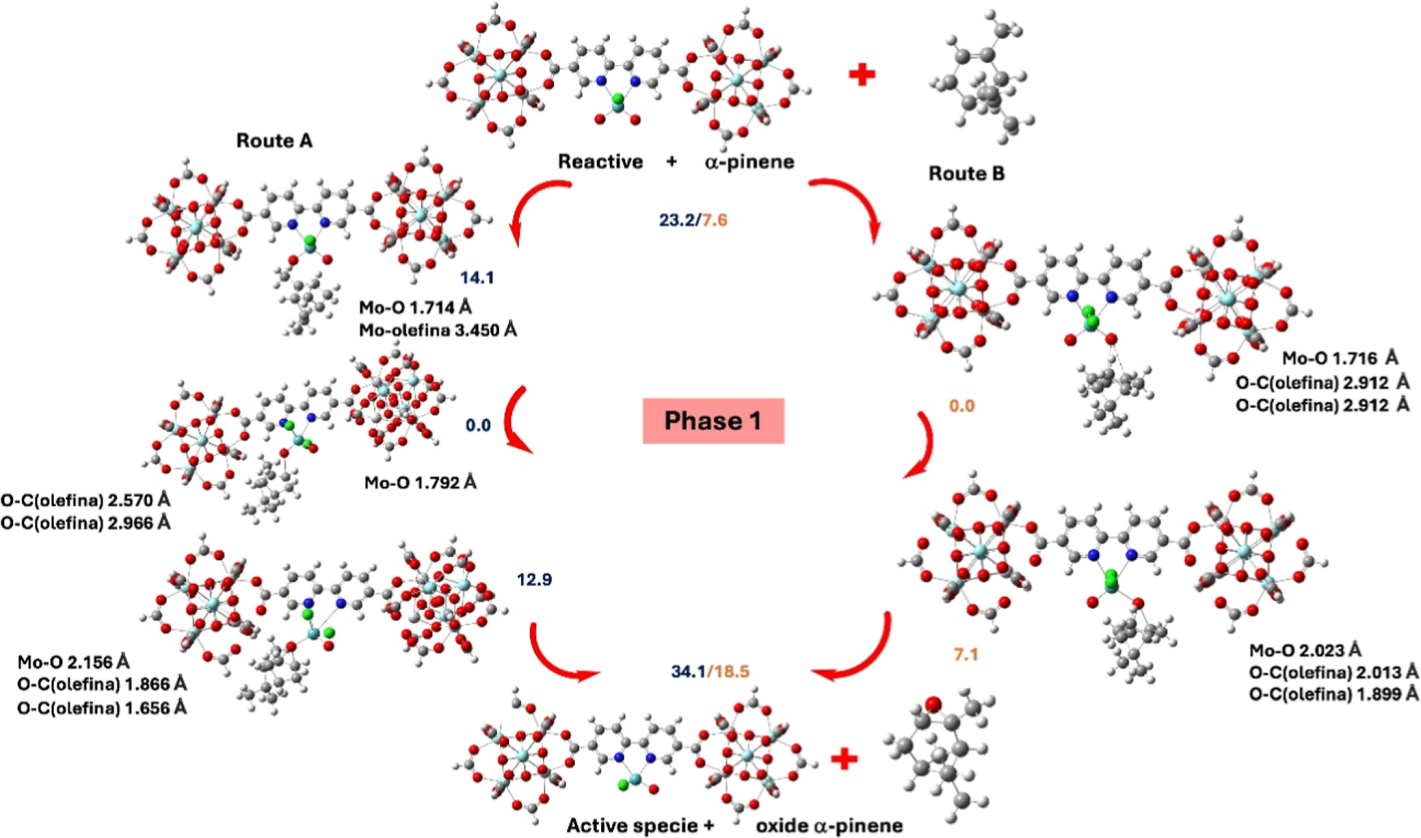
Catalytic cycle and Gibbs energy values associated with phase 1 (one oxygen atom is transferred from the dioxo-Mo center to α-pinene) of the OAT mechanism involving pathways A and B. Values are in kcal/mol.

During phase 2 (dark + O_2_ atmosphere), the EPR technique was used, and a single signal was observed in the spectrum (see Figure 11), attributed to the presence of oxo-Mo^(V)^ ions with a d^1^ configuration. The increase in this signal with time reaches its maximum at 60 min, after which it disappears. This behavior is attributed to the reduction in the catalytic centers from Mo^(VI)^ to Mo^(IV)^, resulting in the formation of a Mo^(V)^ intermediate. The isotropic signal at g_iso_:1.98 (see Figure 1a,b) is found in a region reported for mononuclear Mo^(V)^ complexes, presenting satellites attributed to the isotopes ^95^ Mo (15.7%) and ^97^ Mo (9.5%) with a nuclear spin I: 5/2.^38^ Additionally, the isotropic shape of the signal suggests that paramagnetic species are not magnetically isolated, and that exchange interactions probably occur within neighboring Mo^(V)^ centers.^39^

**Figure 11:**
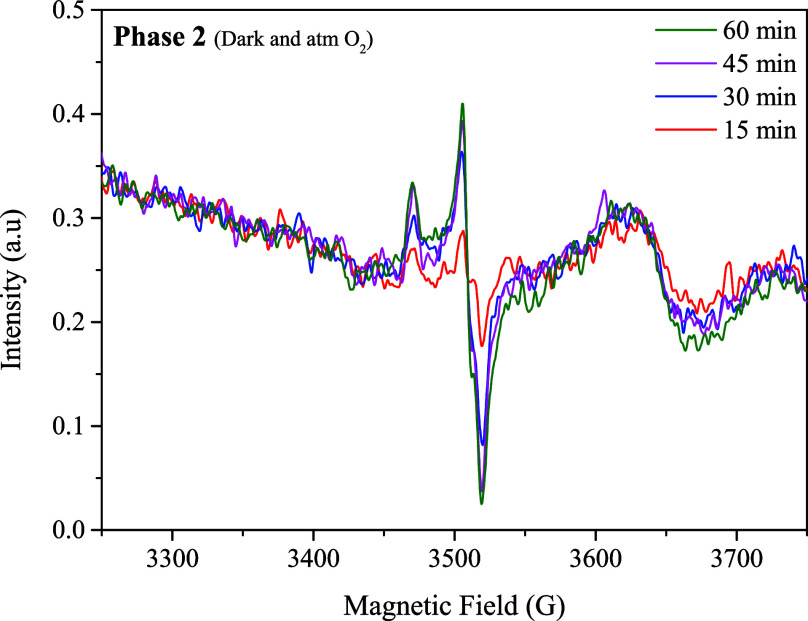
EPR spectrum obtained during the reoxidation (O_2_ + dark), this paramagnetic signal is attributed to the Mo^(V)^ species.

The EPR results are consistent with the computational study of the oxidation of active species that form an oxo-peroxo Mo^(VI)^ species (Figures 12 and 13). The energetic result shows that the reaction tends to form this species. This is a symmetrical structure which presents a Mo=O oxo bond with distance of 1.692 Å and a Mo–O_2_ peroxo bond forming an angle Mo–O–O of 67° and distances Mo–O bond of 1.948 Å and a distance of the O–O bond of 1.414 Å. The formation of this species occurs from Mo^(IV)^ in the reactant passing through a Mo^(V)^ in an anionic Mo-oxo-peroxo intermediate which is the most stable species in this reaction and has been experimentally determined by EPR.^40^ This intermediate structure differs from the Mo^(VI)^ oxo-peroxo species due to a loss of linearity. Specifically, the C–C–O–Ti dihedral angle is 136°, compared to 178° in the parent species. This geometric rearrangement is consequence of the high beta spin density localized over one of the Ti–Ti clusters (Figure 7) generating a modified crystalline structure that show changes in some signals in XRD as 13.1° y 17.1° 2θ mentioned above.^3^ Therefore, the anion Mo-Oxo-peroxo is stabilized by the electronic effects product due to the delocalization of the anionic charge when Mo^(IV)^ is transformed into Mo^(V)^.

**Figure 12:**
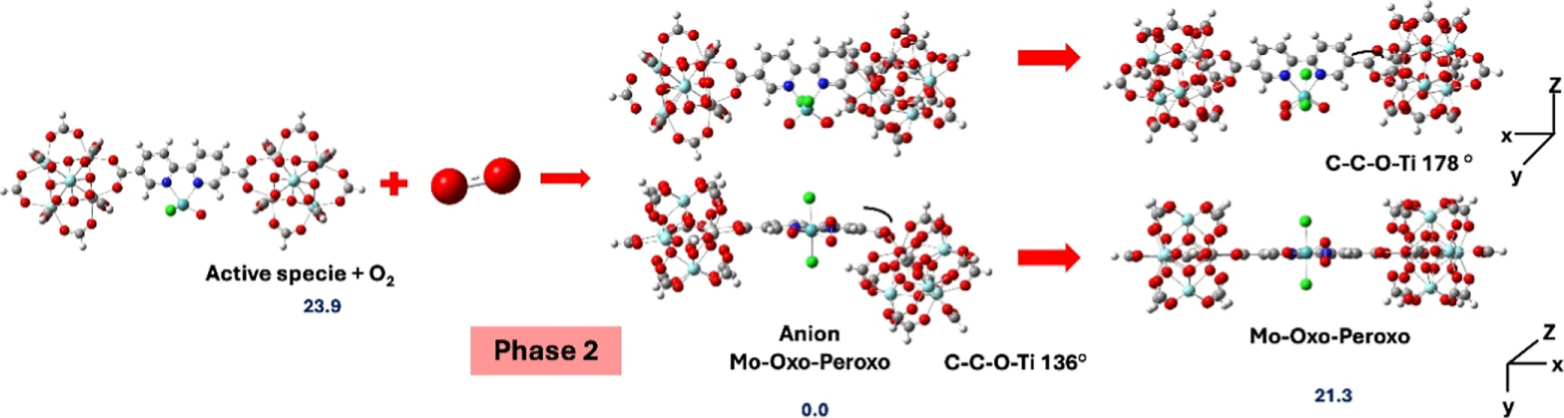
Oxidation by O_2_ to form an oxo-peroxo Mo species and its anionic structure (phase 2: reoxidation of the Mo^(V)^ center by O_2_). Values are relative Gibbs energies in kcal/mol.

**Figure 13:**
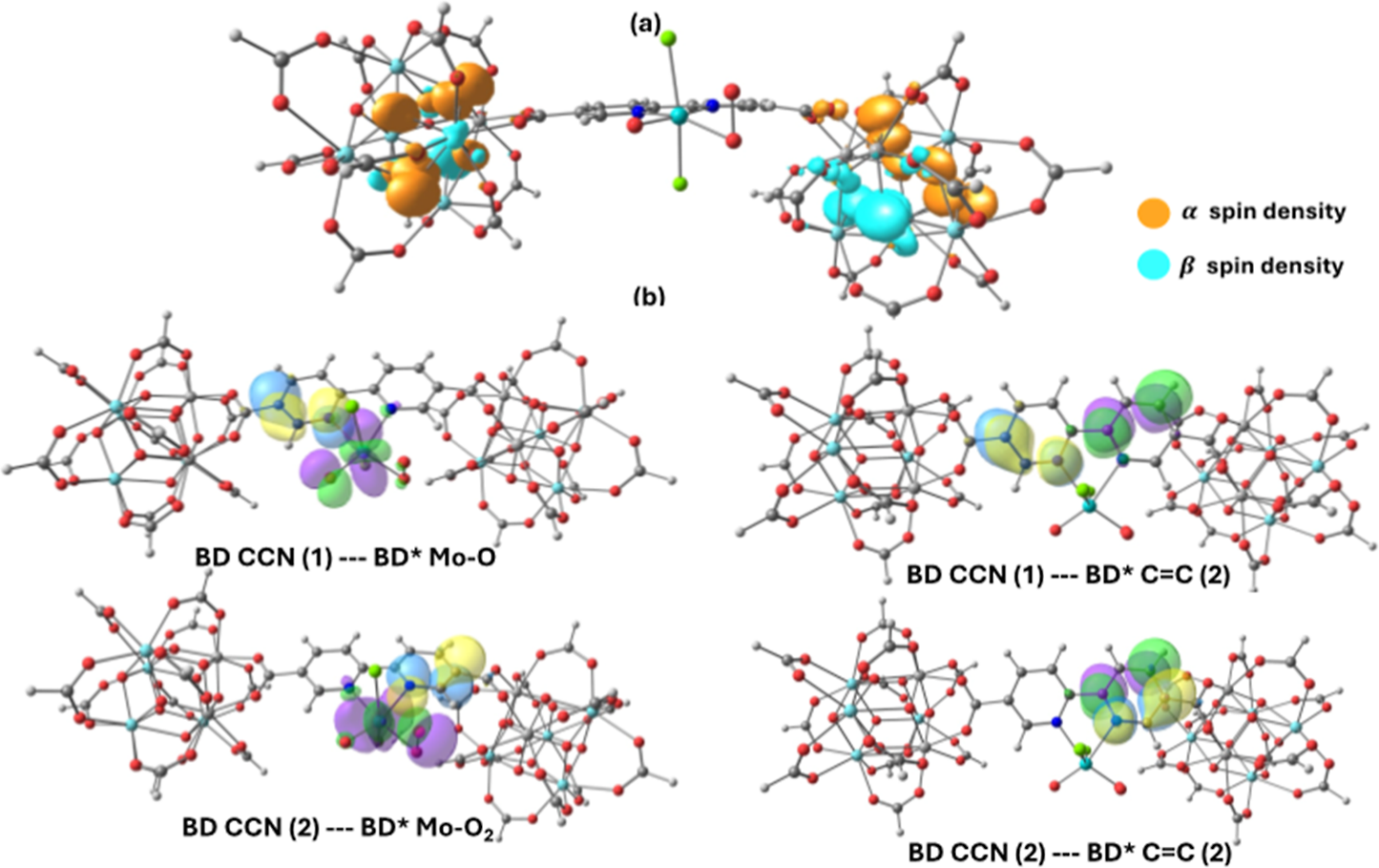
(a) Total spin density of the Mo^(VI)^-Oxo-peroxo anion. Alpha and beta densities are described by yellow and blue contours, respectively. (b) The strongest donor–acceptor interactions in the Mo^(VI)^-Oxo-peroxo anion.

To better understand the electronic structure of the Mo-oxo-peroxo anion, an NBO analysis was carried out to identify the key donor–acceptor interactions. As shown in Figures 12 and 13, the strongest interactions occur between the bonding natural orbitals on the pyridine rings and the antibonding orbitals associated with the Mo–O­(oxo) and Mo–O_2_(peroxo) bonds. This indicates that the delocalized charge, initially distributed over the aromatic rings, is transferred to the MoO_2_Cl_2_ unit, which helps explain the high reactivity at this catalytic center. Additionally, the analysis suggests that this delocalized charge may also be transferred to other antibonding orbitals on the pyridine rings, pointing to the presence of intraligand charge transfer and broadening the potential applications of this system.

In phase 3 of the OAT reaction (350 nm irradiation under Ar), the EPR spectrum displayed the same single isotropic radical signal observed previously (see Figure 14). This signal is consistent with the formation of oxo-Mo^(V)^ species, produced through the reduction of Mo^(VI)^ to Mo^(IV)^ during the OAT process. The computational study of the Mo-Oxo-peroxo anion shows the reaction with α-pinene through a direct attack on the olefin double bond at one of the peroxo group oxygens, yielding a Mo–O–O–O_2_-pinene intermediate with an O–C bond at 1432 Å. In this intermediate, the Mo–O_2_ forms an angle Mo–O–O of 124° generating a longer Mo–O distance (3.015 Å) than in the anion. Also, between the olefin and the MoCl_2_OO_2_ group, the dihedral angles Mo–O–O–C and O–O–C–C are generated to 160° and 51°, respectively. These rearrangements give rise to a more stable intermediate **Mo**–**O**–**O**–**C**–**C** in which the O–O–C–C dihedral angle changes to 5°, generating a cyclic structure with distances of 1.583 and 1.545 Å for the O–C and C–C bonds, respectively. In this intermediate state, the distances between the oxygen atoms and the molybdenum are 2.111 Å and 3.136 Å, respectively. This suggests that the furthest oxygen atom is bound to the α-pinene oxide, while the shortest distance allows the reactant to regenerate. Thus, this intermediate cyclic suffers a diagonal break producing the α-pinene oxide and regenerating the reactant, which continues the heterogeneous reaction until the total consumption. Figure 15 illustrates the catalytic cycle. The energy profile shows that the product is the most stable species, followed by the cyclic **Mo**–**O**–**O**–**C**–**C** intermediate and the **Mo**–**O**–**O**_2_–**pinene** intermediate, with relative energies of 5.0 and 11.5 kcal/mol, respectively. Moreover, phases 2 and 3 progress toward product formation rather than revert to the reactants, confirming the efficiency of the catalyst.

**Figure 14:**
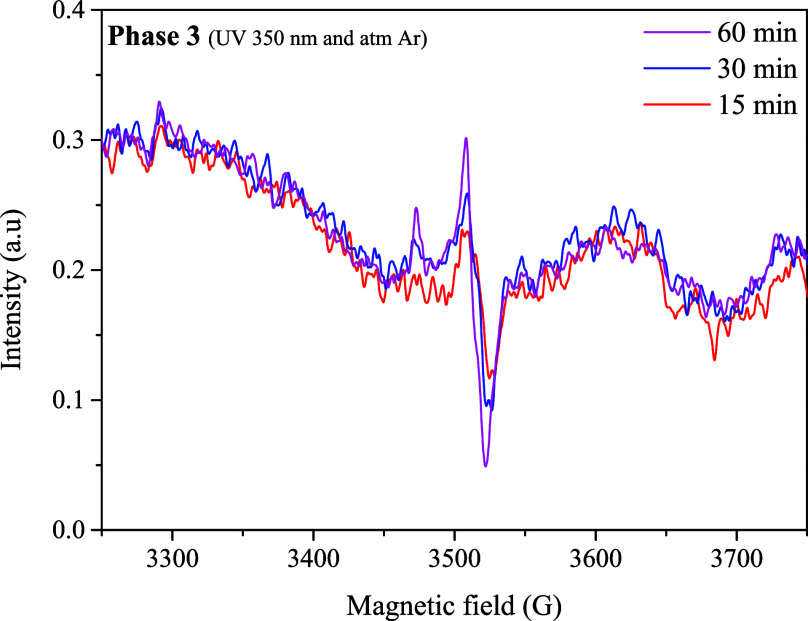
EPR spectrum obtained during phase 3 of the OAT reaction (Ar + 350 nm), this paramagnetic signal is attributed to the Mo^(V)^ species.

**Figure 15:**
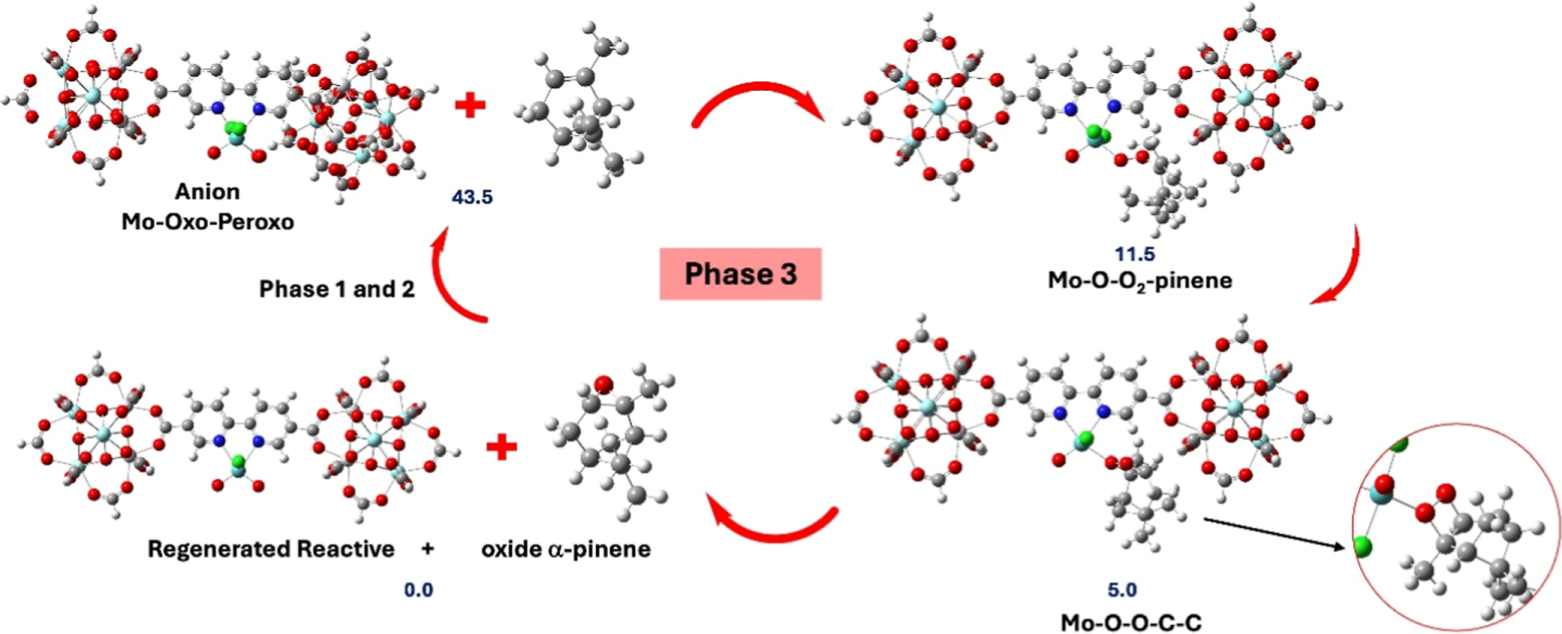
Representative catalytic cycle and Gibbs energy values associated with phase 2 (reoxidation of the Mo^(V)^ center by O_2_ to the former Mo^(VI)^–O–O peroxo) and 3 (OAT from the molybdenum oxo-peroxo center to α-pinene) of the OAT mechanism. Values are in kcal/mol.

## Conclusions

Combined structural analysis using theoretical simulations and experimental techniques (XRD and XPS) confirmed the formation of both the (Zr)-UiO-67-(bpdc,bpydc) and (Ti/Zr)-UiO-67-(bpdc,bpydc) frameworks. These results show that Ti-substitution maintains the crystalline structure, while markedly enhancing photoactivity and electronic stability. Catalytically, phase 1 proceeds most favorably through route B, which involves a direct attack of the olefin’s double bond on the oxo group of the active Mo center. In phase 2, a key Mo^(V)^ intermediate was identified through DFT calculations and experimentally verified by EPR spectroscopy. Together, these findings support a consistent oxygen-atom-transfer (OAT) mechanism that reflects the structural and redox characteristics of the system. Overall, the strong interplay between theoretical modeling and experimental validation provides a powerful framework for understanding functionalized MOFs and guides the rational design of heterogeneous catalysts with well-defined active sites. This integrated strategy can be applied to other hybrid systems, enabling performance optimization and accelerating their use in sustainable catalytic processes.

## Data Availability

The data underlying this study are available in the published article.

## References

[1] Kim M., Cahill J. F., Fei H., Prather K. A., Cohen S. M., Su Y., Prather K. A., Cohen S. M., Burrows A. D., Kim M., Cahill J. F., Fei H., Prather K. A., Cohen S. M. (2012). Postsynthetic
Ligand and Cation Exchange in Robust Metal-Organic Frameworks. J. Am. Chem. Soc..

[2] Wang A., Zhou Y., Wang Z., Chen M., Sun L., Liu X. (2016). Titanium incorporated
with UiO-66­(Zr)-type Metal-Organic Framework
(MOF) for photocatalytic application. RSC Adv..

[3] Hendon C. H., Tiana D., Fontecave M., Sanchez C., D’arras L., Sassoye C., Rozes L., Mellot-Draznieks C., Walsh A. (2013). Engineering the optical response of the titanium-MIL-125 metal-organic
framework through ligand functionalization. J. Am. Chem. Soc..

[4] Kaskel, S. Group 4 Metals as Secondary Building Units: Ti, Zr, and Hf-based MOFs. In The Chemistry of Metal-Organic Frameworks: Synthesis, Characterization, and Applications; Wiley-VCH Verlag GmbH & Co. KGaA, 2016; pp 137–170. 10.1002/9783527693078.ch6.

[5] Navarro
Amador R., Carboni M., Meyer D. (2017). Sorption and photodegradation
under visible light irradiation of an organic pollutant by a heterogeneous
UiO-67-Ru-Ti MOF obtained by post-synthetic exchange. RSC Adv..

[6] Nickerl G., Leistner M., Helten S., Bon V., Senkovska I., Kaskel S. (2014). Integration of accessible secondary
metal sites into
MOFs for H2S removal. Inorg. Chem. Front..

[7] Valdivieso
Zárate L., Bravo-Sanabria C. A., Ramírez-Caballero G. E., Martinez Ortega F. (2023). Photoinduced Oxygen Atom Transfer to α-pinene
and R-carvone using a Dioxo-Molybdenum (VI) Complex Incorporated within
a Modified UiO-67 (Zr/Ti) MOF. Eur. J. Inorg.
Chem..

[8] Martínez H., Cáceres M. F., Martínez F., Páez-Mozo E. A., Valange S., Castellanos N. J., Molina D., Barrault J., Arzoumanian H. (2016). Photo-epoxidation
of cyclohexene, cyclooctene and 1-octene
with molecular oxygen catalyzed by dichloro dioxo-(4,4′-dicarboxylato-2,2′-bipyridine)
molybdenum­(VI) grafted on mesoporous TiO2. J.
Mol. Catal. A Chem..

[9] Teruel H., Sierralta A. (1996). Dioxomolybdenum­(VI)
complexes: Relations between oxygen
transfer and 95Mo NMR chemical shift. J. Mol.
Catal. A Chem..

[10] Neves P., Gomes A. C., Amarante T. R., Paz F. A. A., Pillinger M., Gonçalves I. S., Valente A. A. (2015). Incorporation of a dioxomolybdenum­(VI)
complex in a ZrIV-based Metal-Organic Framework and its application
in catalytic olefin epoxidation. Microporous
Mesoporous Mater..

[11] Liimatainen J., Lehtonen A., Sillanpaa R. (2000). cis-Dioxomolybdenum­(VI)
complexes
with tridentate and tetradentate Schiff base ligands. Preparation,
structures and inhibition of aerial oxidation of aldehydes. Polyhedron.

[12] Øien, S. Synthesis and Characterization of Modified UiO-67 metal-organic Frameworks (Thesis for the Master’s Degree in Chemistry); UNIVERSITY OF OSLO, 2012.

[13] Leus K., Liu Y. Y., Van Der Voort P. (2014). Metal-organic frameworks as selective
or chiral oxidation catalysts. Catal. Rev. -
Sci. Eng..

[14] Jain A., Ong S. P., Hautier G., Chen W., Richards W. D., Dacek S., Cholia S., Gunter D., Skinner D., Ceder G., Persson K. A. (2013). Commentary:
The materials project:
A materials genome approach to accelerating materials innovation. APL Mater..

[15] Gražulis S., Daškevič A., Merkys A., Chateigner D., Lutterotti L., Quirós M., Serebryanaya N. R., Moeck P., Downs R. T., Le Bail A. (2012). Crystallography Open
Database (COD): An open-access collection of crystal structures and
platform for world-wide collaboration. Nucleic
Acids Res..

[16] Groom C. R., Bruno I. J., Lightfoot M. P., Ward S. C. (2016). The Cambridge Structural
Database. Acta Crystallogr. B Struct. Sci. Cryst.
Eng. Mater..

[17] Palmer, D. C. Crystalmaker. Crystalmaker Software Ltd, Pegbroke, England, 2014.

[18] Momma K., Izumi F. (2008). VESTA: a three-dimensional visualization
system for electronic and
structural analysis. J. Appl. Crystallogr..

[19] Kühne T. D., Iannuzzi M., Del Ben M., Rybkin V. V., Seewald P., Stein F., Laino T., Khaliullin R. Z., Schütt O., Schiffmann F., Golze D., Wilhelm J., Chulkov S., Bani-Hashemian M. H., Weber V., Borštnik U., Taillefumier M., Jakobovits A. S., Lazzaro A., Pabst H., Müller T., Schade R., Guidon M., Andermatt S., Holmberg N., Schenter G. K., Hehn A., Bussy A., Belleflamme F., Tabacchi G., Glöß A., Lass M., Bethune I., Mundy C. J., Plessl C., Watkins M., VandeVondele J., Krack M., Hutter J. (2020). CP2K: An electronic
structure and molecular dynamics software package - Quickstep: Efficient
and accurate electronic structure calculations. J. Chem. Phys..

[20] Goedecker S., Teter M., Hutter J. (1996). Separable
dual-space Gaussian pseudopotentials. Phys.
Rev. B Condens. Matter Mater. Phys..

[21] VandeVondele J., Hutter J. (2007). Gaussian basis sets for accurate
calculations on molecular
systems in gas and condensed phases. J. Chem.
Phys..

[22] VandeVondele J., Krack M., Mohamed F., Parrinello M., Chassaing T., Hutter J. (2005). Quickstep: Fast and accurate density
functional calculations using a mixed Gaussian and plane waves approach. Comput. Phys. Commun..

[23] Grimme S., Antony J., Ehrlich S., Krieg H. (2010). A consistent and accurate
ab initio parametrization of density functional dispersion correction
(DFT-D) for the 94 elements H-Pu. J. Chem. Phys..

[24] Grimme S., Ehrlich S., Goerigk L. (2011). Effect of the damping function in
dispersion corrected density functional theory. J. Comput. Chem..

[25] Tian C., Zhao J., Ou X., Wan J., Cai Y., Lin Z., Dang Z., Xing B. (2018). Enhanced Adsorption
of p -Arsanilic
Acid from Water by Amine-Modified UiO-67 as Examined Using Extended
X-ray Absorption Fine Structure, X-ray Photoelectron Spectroscopy,
and Density Functional Theory Calculations. Environ. Sci. Technol..

[26] Hu P., Wang R., Gao Z., Jiang S., Zhao Z., Ji H., Zhao Z. (2021). Improved interface
compatibility of hollow H-Zr0.1Ti0.9O2
with UiO-66-NH2 via Zr-Ti bidirectional penetration to boost visible
photocatalytic activity for acetaldehyde degradation under high humidity. Appl. Catal., B.

[27] Zhou Y., Liu J., Long J. (2021). Journal of
Solid State Chemistry Photocatalytic oxidation
5-Hydroxymethylfurfural to 2, 5-diformylfuran under air condition
over porous TiO 2 @ MOF. J. Solid State Chem..

[28] Zhou Y., Liu J., Long J. (2021). Photocatalytic oxidation
5-Hydroxymethylfurfural to
2, 5-diformylfuran under air condition over porous TiO2@MOF. J. Solid State Chem..

[29] Hu P., Wang R., Gao Z., Jiang S., Zhao Z., Ji H., Zhao Z. (2021). Improved interface
compatibility of hollow H-Zr0.1Ti0.9O2
with UiO-66-NH2 via Zr-Ti bidirectional penetration to boost visible
photocatalytic activity for acetaldehyde degradation under high humidity. Appl. Catal., B.

[30] Braglia L., Borfecchia E., Lomachenko K. A., Bugaev A. L., Guda A. A., Soldatov A. V., Bleken B. T. L., Øien-ØDegaard S., Olsbye U., Lillerud K. P., Bordiga S., Agostini G., Manzoli M., Lamberti C. (2017). Tuning Pt and Cu sites population
inside functionalized UiO-67 MOF by controlling activation conditions. Faraday Discuss..

[31] Liu J., Huang X., Jia L., Liu L., Nie Q., Tan Z., Yu H. (2023). Microwave-Assisted
Rapid Substitution of Ti for Zr
to Produce Bimetallic (Zr/Ti)­UiO-66-NH2 with Congenetic “Shell–Core”
Structure for Enhancing Photocatalytic Removal of Nitric Oxide. Small.

[32] Lazar A., Thiel W. R., Singh A. P. (2014). Synthesis
and characterization of
3-[N,N′-bis-3-(salicylidenamino) ethyltriamine] Mo­(vi)­O2@SBA-15:
A highly stable and reusable catalyst for epoxidation and sulfoxidation
reactions. RSC Adv..

[33] Samat M. H., Ali A. M. M., Taib M. F. M., Hassan O. H., Yahya M. Z. A. (2016). Hubbard
U calculations on optical properties of 3d transition metal oxide
TiO2. Results Phys..

[34] Imanova G. T., Kaya M. (2021). Structural Analysis of Nanoparticle
Zirconium Dioxide: A Comprehensive
Review. Modern Approaches on Material Science
05.

[35] Lu X., Deng L., Kerisit S., Du J. (2018). Structural role of
ZrO2 and its impact on properties of boroaluminosilicate nuclear waste
glasses. npj Mater. Degrad..

[36] Amini M., Haghdoost M. M., Bagherzadeh M. (2013). Oxido-peroxido molybdenum­(VI) complexes
in catalytic and stoichiometric oxidations. Coord. Chem. Rev..

[37] Valdivieso
Zarate L. M., Bravo Sanabria C. A., Ramírez Caballero G. E., Martínez Ortega F. (2023). Photoinduced Oxygen Atom Transfer
to α-Pinene and R-Carvone using a Dioxo-Molybdenum (VI) Complex
Incorporated within a Modified UiO-67 (Zr/Ti) MOF. Eur. J. Inorg. Chem..

[38] Heinze K., Fischer A. (2010). Oxidomorybdenum­(IV), -(V), -(VI) complexes with relevance
to molybdenum enzymes: Oxygen atom transfer, redox chemistry and EPR
spectroscopy. Eur. J. Inorg. Chem..

[39] Martinez
Quiñonez H., Amaya A. ´.A., Paez-Mozo E. A., Martinez Ortega F. (2022). Aminothiazole Ligand-Type Dioxo-Mo­(VI) Complex Anchored
on TiO2 Nanotubes for Selective Oxidation of Monoterpenes with Light
and O2. Top. Catal..

[40] Martínez
Q H., Valezi D. F., Di Mauro E., Páez-Mozo E. A., Martínez O F. (2022). Characterization of peroxo-Mo and superoxo-Mo intermediate
adducts in Photo-Oxygen Atom Transfer with O2. Catal. Today.

